# ANCAC: amino acid, nucleotide, and codon analysis of COGs – a tool for sequence bias analysis in microbial orthologs

**DOI:** 10.1186/1471-2105-13-223

**Published:** 2012-09-08

**Authors:** Arno Meiler, Claudia Klinger, Michael Kaufmann

**Affiliations:** 1 The Protein Chemistry Group, Institute for Medical Biochemistry, Centre for Biomedical Education and Research, School of Medicine, Faculty of Health, Witten/Herdecke University, Stockumer Str. 10, Witten, 58448, Germany

## Abstract

**Background:**

The COG database is the most popular collection of orthologous proteins from many different completely sequenced microbial genomes. Per definition, a cluster of orthologous groups (COG) within this database exclusively contains proteins that most likely achieve the same cellular function. Recently, the COG database was extended by assigning to every protein both the corresponding amino acid and its encoding nucleotide sequence resulting in the NUCOCOG database. This extended version of the COG database is a valuable resource connecting sequence features with the functionality of the respective proteins.

**Results:**

Here we present ANCAC, a web tool and MySQL database for the analysis of amino acid, nucleotide, and codon frequencies in COGs on the basis of freely definable phylogenetic patterns. We demonstrate the usefulness of ANCAC by analyzing amino acid frequencies, codon usage, and GC-content in a species- or function-specific context. With respect to amino acids we, at least in part, confirm the cognate bias hypothesis by using ANCAC’s NUCOCOG dataset as the largest one available for that purpose thus far.

**Conclusions:**

Using the NUCOCOG datasets, ANCAC connects taxonomic, amino acid, and nucleotide sequence information with the functional classification via COGs and provides a GUI for flexible mining for sequence-bias. Thereby, to our knowledge, it is the only tool for the analysis of sequence composition in the light of physiological roles and phylogenetic context without requirement of substantial programming-skills.

## Background

Within the COG database, orthologous protein sequences, *i. e.* sequences assumed to achieve the same biochemical function, are assigned to individual clusters [1-4]. Although more than thousand microbial genomes are completely sequenced by now, the current COG database containing only 66 representatives still plays an important role in genomics, especially when functional aspects of proteins are taken into consideration [5]. In addition to the classical COG, the arCOG database has been constructed representing a refinement with respect to 41 archaeal genomes [6]. Both databases originally contained no sequence information directly connected with protein names. This drawback was recently eliminated by the construction of NUCOCOG, the nucleotide sequences containing COG database [7]. Thus, NUCOCOG allows linking sequence signatures of both amino acid and nucleotide sequences with functional aspects of the corresponding proteins. The database consists of three XML-files: nucocog.xml containing the classical COG-database, arnucocog.xml containing the arCOG database, and nucocog2.xml, containing a non-redundant combination of both the classical COG and arCOG databases. This report describes ANCAC, a web-tool capable of mining the NUCOCOG database with respect to the frequencies of amino acids, nucleotides and codons within COG sequences. The user selects i) the database, ii) the type of sequence feature to be analyzed (amino acid, nucleotide or codon), iii) a sequence feature or a set of sequence features of that type, and iv) a set of organisms the sequences of which are to be considered. The web-interface then calculates the absolute and relative abundances of the selected feature(s) in the sequences of each COG and returns a ranking with respect to the calculated abundance indexes. To demonstrate the usefulness of ANCAC, we confirmed earlier findings about correlations between sequence composition and growth temperature or oxygen metabolism as well as parts of the “cognate bias hypothesis” [8] which states that early in evolutionary history the biosynthetic enzymes for amino acid x gradually lost residues of x, thereby reducing the threshold for deleterious effects of x scarcity [9].

## Implementation

### Overall architecture

As a framework to analyze sequence features of both amino acid and nucleotide sequences derived from COGs we constructed a MySQL database and transferred to it all datasets of the NUCOCOG, arNUCOCOG, and NUCOCOG2 databases [7] using Perl-scripts. For each individual sequence the sequence length, the numbers of each amino acid, each nucleotide and each codon were pre-calculated and stored in additional database tables. In addition, hierarchical taxonomic data were extracted from the NCBI taxonomy database [10] and included in a separate table. A web-interface was set up using the Apache2 web-server, Perl-CGI, and Perl-DBI for SQL database connection. User-interaction was implemented client-side in JavaScript and jQuery including the plugins jqPlot, DataTables, and jsTree. The GUI was designed as a horizontal file tab menu partitioning the different input options and the output.

### Input sections

**Figure 1:**
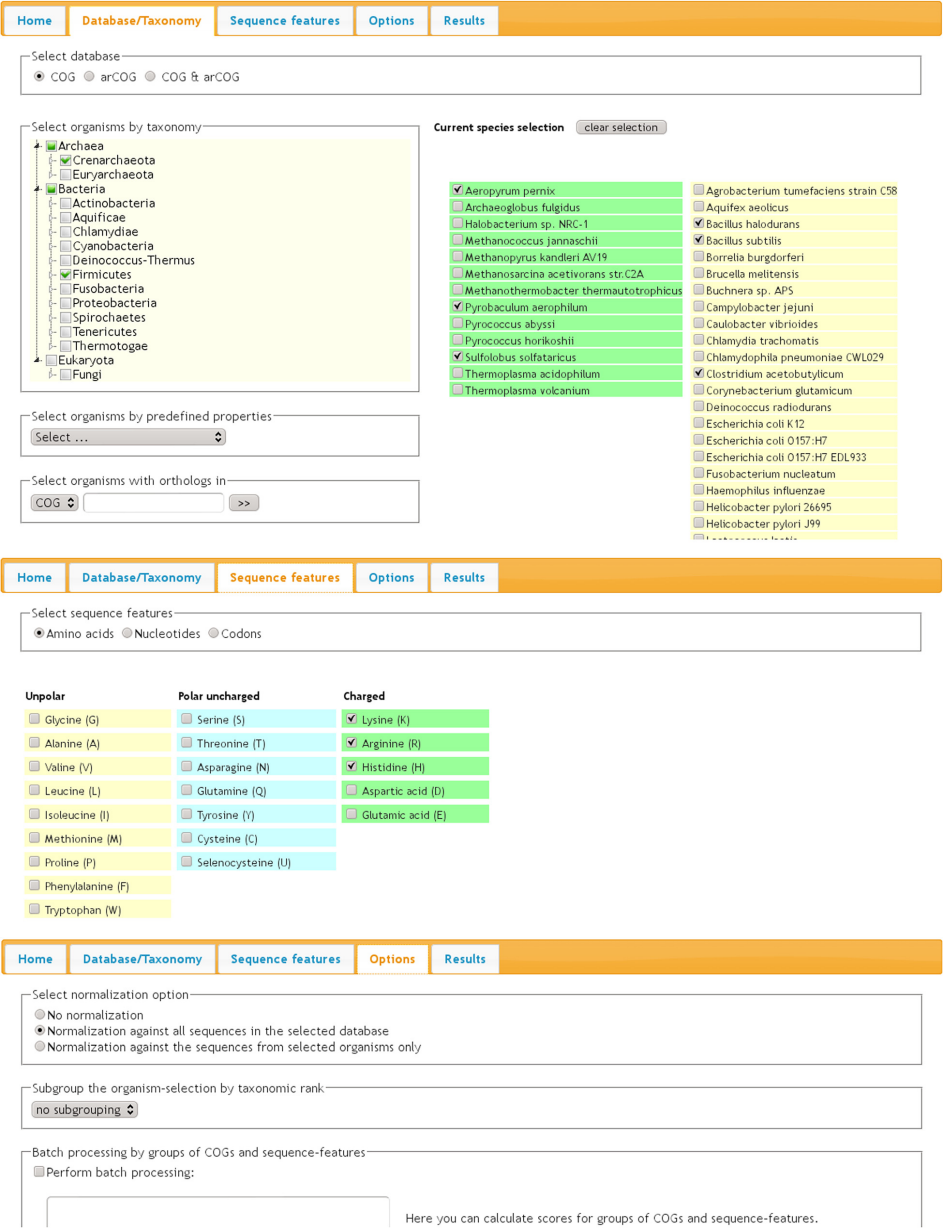
**Input sections.** Screenshots of excerpts of the three main input sections “Database/Taxonomy”, “Sequence features”, and “Options” are presented.

The kind of analysis to be performed is determined via selections in the three input tabs. Screenshots of all of these are assembled in Figure 1.

#### Database/Taxonomy

The user starts here by specifying which database, *i. e.* COG, arCOG or both, should be the basis of the analysis. Depending on the chosen database a selection of available organisms is displayed. Patterns of organisms to be analyzed can then be specified by either manually selecting particular microorganisms of interest or by making use of one of three input aids provided by the tool. Here, groups of organisms can be selected i) by taxon on any level using a hierarchical taxonomic tree, ii) by traits or environmental conditions using pre-defined patterns or iii) by the occurrence of organisms in a COG of choice. Selections by several criteria can be made consecutively in order to form a superset of organisms, *i. e.* by logical AND operations.

#### Sequence features

In this tab, the type of sequence feature is selected, *i. e.* amino acid, nucleotide or codon. Subsequently, any set of features of that type can be defined.

#### Options

Finally, the user may affect both the computation and the output by making selections in the “Options” tab. Without normalization the tool calculates the average percentage of the selected sequence features (APSF) in each individual COG or alternatively in sequence subsets of each individual COG if taxonomic sub-grouping is selected. Alternatively, one of the two options of normalization may be chosen in order to get relative frequency scores.

i) “Normalize against all sequences in the selected database” determines a frequency score by dividing the APSF by the average percentage of the selected features calculated for all sequences within the whole selected database.

ii) “Normalize against the sequences from selected organisms only” determines the frequency score by dividing the APSF by the average percentage of the selected features calculated for the sequences within the selected organisms only.

By default one score is calculated for each COG using the sequences of all selected organisms in this COG. In order to add taxonomic resolution “subgroup the organism-selection by taxonomoc rank” can be chosen assigning sequences to groups along the taxonomic level of choice. Thus, one COG score will be calculated for each taxon occurring in the organism selection at the specified level.

A different mode of analysis from what has been described above is provided by “Batch processing by groups of COGs and sequence-features”. Here, the user can precisely define queries by text input obeying simple formatting rules. This allows calculating scores for any combination of sequence features within selected sets of COGs. Most importantly, cumulative scores for user-defined groups of COGs allow the detection of sequence bias in arbitrary biological contexts such as biochemical pathways or cellular location.

### Computations

The server calculates a score for the sequences of each COG or optionally each group of COGs in case of batch processing. Only sequences derived from the organisms that have been selected are considered for computation. The APSFs are calculated by summing up pre-calculated feature counts and sequence lengths which are stored in the database for each sequence separately. To interpret sequence bias, the frequencies obtained are optionally normalized as described above.

### Output section

**Figure 2:**
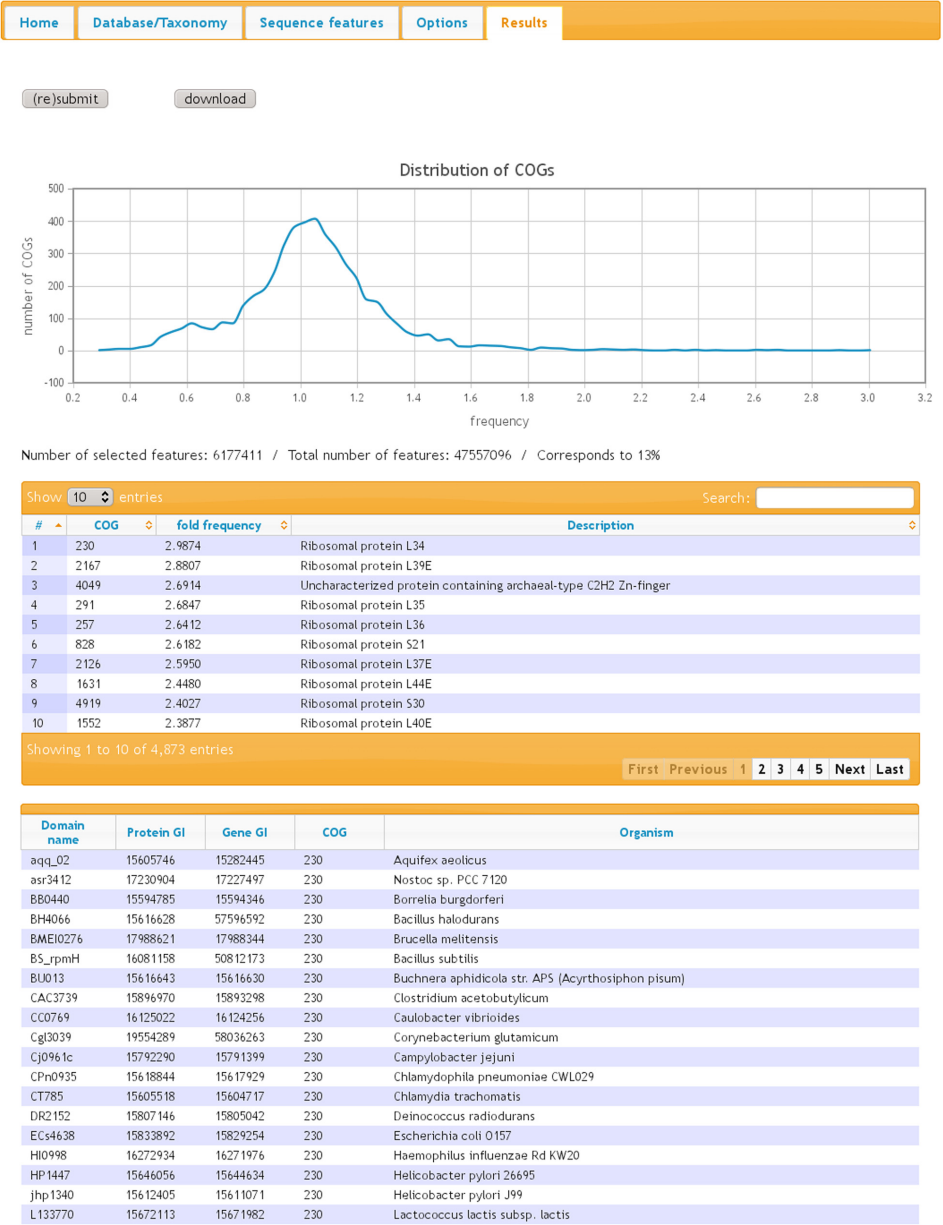
**Output section.** Screenshot of the “Results” tab of ANCAC. The ranking for COGs where positively charged amino acids are overrepresented is shown. Input parameters: COG database, all organisms, Amino acids Lysine (K)/Arginine (R)/Histidine (H), normalization against all sequences in the selected database. COG 230 as the high scoring COG was clicked resulting in a summary at the bottom of the output page showing all sequences from COG 230 that were analyzed.

On “(re)submit” in the “Results” tab computation is performed and a table of results as well as a graph are displayed (Figure 2). The graph visualizes the distribution of COG scores. The table presents i) a consecutive number, ii) the COG number, iii) the calculated frequency score, and iv) the functional description of the COG. By clicking on a column header in the table, the results are arranged according to the elements within the respective column, either ascending or descending. The output can be filtered and browsed for any keyword or value by using the search field on top of the table. After clicking on a COG number, all sequences considered for the calculation of the respective score are displayed in a separate table including their domain name, protein GI-number, gene GI-number, COG number, and name of the source organism. The table of results can also be downloaded as a tab-separated text file. In case of using a normalization option in addition to individual frequency scores, the “Number of selected features”, the “Total number of features”, and the corresponding average percentage of the selected features are displayed.

## Results and discussion

ANCAC is a tool for analyzing the sequences in COGs in a functional and phylogenetic context by allowing the user to freely determine organisms and sequence features in any possible combination. In order to demonstrate the power of ANCAC and to make use of its larger pool of organisms and sequences we now briefly re-examine relationships between sequence features and protein function or sequence signatures and biological context already published, however without any claim to completeness.

### Positively charged amino acids and ribosomal proteins

The integrity of the ribosome is based on the complex interaction between ribosomal proteins and ribosomal RNA. Here electrostatic interactions between numerous arginine and lysine residues, particularly those in tail extensions, and the phosphate groups of the RNA backbone mediate many protein-RNA contacts [11]. Ranking the COGs of all species contained in the COG database according to their relative abundance of positively charged amino acids (K, H, and R) almost exclusively yields top-scores for COGs containing ribosomal proteins, demonstrating the power of ANCAC to link sequence composition with protein function (Figure 2).

### Cognate bias hypothesis

**Figure 3:**
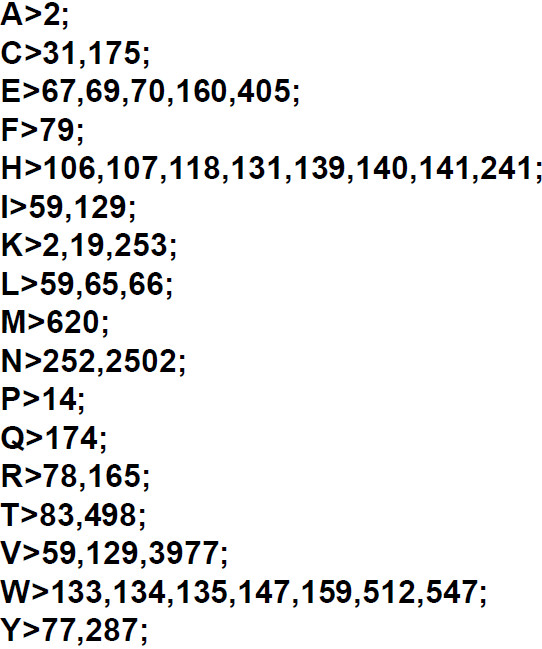
**Exemplary input for batch processing.** Shown is the query for the analysis of the cognate bias hypothesis, each line stating an amino acid, and the list of COG numbers exclusively associated with the biosynthetic pathway. One score for each line will be calculated.

**Figure 4:**
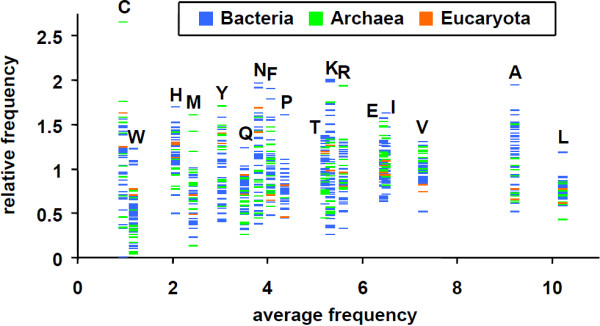
**Cognate bias hypothesis I: data retrieved via ANCAC.** Relative frequency of amino acids in their biosynthetic* enzymes plotted against their frequency in all protein sequences of the COG database. Each data point represents the relative frequency score calculated from the sequences of one species. Database: COG, organisms: all, sequence features: amino acids, normalization: against all sequences in the selected database, subgrouping: by species.

**Figure 5:**
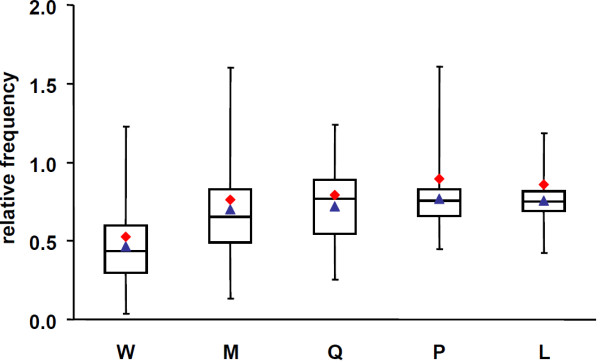
**Cognate bias hypothesis II: ANCAC versus Perlstein*et al.*** Boxplot showing the distribution of relative frequencies of the 5 underrepresented amino acids from Figure 4. The blue triangles represent the average of the distribution. The red squares indicate the averages as determined by Perlstein *et al.*[9].

The cognate bias hypothesis stating that an amino acid is underrepresented in the biosynthetic enzymes for its own synthesis has been tested by Perlstein *et al.* for the genomes of *E. coli*, *B. subtilis*, *M. jannaschii*, *S. cerevisiae*, and *H. sapiens*[9]. The cognate bias hypothesis is an excellent example for the need to link biochemical function to sequence composition. Figure 3 shows the input of single ANCAC query in batch processing mode with taxonomic subgrouping by species. As can be seen in the graphical representation of the output (Figure 4), the validity of the cognate bias hypothesis depends on both the amino acid and organism (or group of organisms) under investigation. We have compared the data published by Perlstein *et al.* to those obtained by ANCAC (Figure 5). With respect to all five amino acids emphasized by Perlstein *et al.*, a slightly more pronounced mean underrepresentation could be detected by ANCAC. This might be attributed to the fact that Perlstein *et al*. as one of only 5 organisms have included *H. sapiens* that shows only weak bias. In addition there are differences in the number of organisms under investigation (66 in this report versus 5 in [9]), the enzymes assigned to a certain biosynthetic pathway (Figure 4 in this report versus not stated in [9]), and the chosen reference (all sequences of the whole COG database in this report versus all sequences involved in amino acid biosynthetic pathways only in [9]).

### Amino acid composition and growth temperature

**Figure 6:**
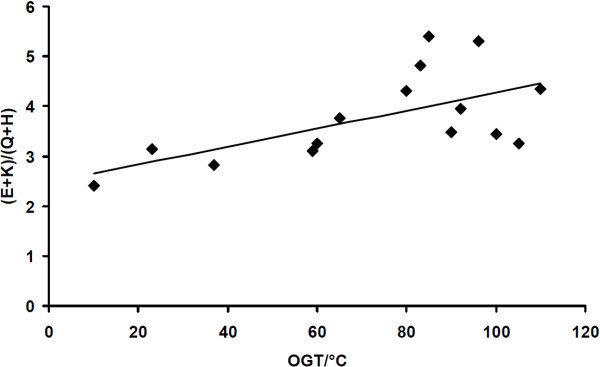
**Correlation between OGTs and amino acid composition in Archaea.** A plot of the OGTs of the Archaeae in the arCOG database versus their (E + K)/(Q + H) ratios is presented confirming the correlation described by Farias and Bonato [12].

Compared to their mesophilic counterparts, thermophilic and hyperthermophilic proteins contain more glutamates (E) and lysines (K) but less glutamines (Q) and histidines (H). There is even a correlation between the ratio (E + K)/(Q + H) in proteomes of different organisms and their respective optimum growth temperature (OGT) [12]. Figure 6 shows a confirmation of this correlation for *Archaeae* using the arCOC database by plotting the OGTs as published by Makarova *et al.*[6] versus the computed (E + K)/(Q + H) ratios as determined by ANCAC. Assuming a linear relationship, a slope of 0.02°C^-1^ of the regression line (r = 0.61) was obtained.

### **Codon usage and growth temperature**

**Figure 7:**
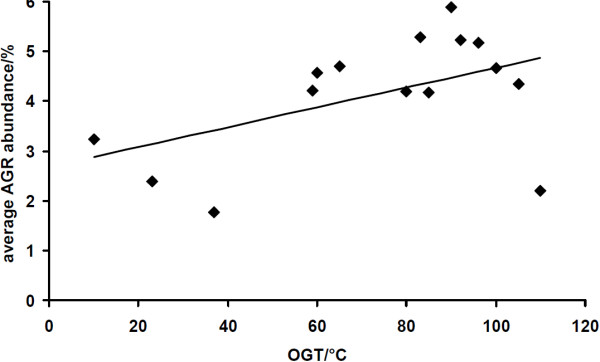
**Correlation between OGTs and codon usage for arginine in Archaea.** A plot of the OGTs of the Archaeae in the arCOG database versus their relative abundances of AGR codons for arginine is presented confirming the correlation described by Van der Linden and Farias [13].

Not only differences in amino acid composition but also differences in codon usage have been described. Here mesophilic organisms were compared to thermophilic or hyperthermophilic microbes. For instance, the preferential usage of AGR codons (R = puRine = A or G) for arginine in thermophiles and hyperthermophiles has been reported [13]. A verification of these findings is shown in Figure 7 where the OGTs of all organisms of the arCOG database are plotted against the average abundance in AGR codons in their genomes. Assuming a linear relationship, a slope of 0.02%°C^-1^ of the regression line (r = 0.49) was obtained.

### GC content and aerobiosis

Naya *et al.* reported that aerobic prokaryotes display a significant increment in genomic GC content in relation to anaerobic ones [14]. Querying the COG database by ANCAC and selecting the pre-defined patterns “Aerobia” and “Anaerobia” we verify these findings conveniently resulting in 53.94% and 44.78% in genomic GC, respectively.

### Future prospects

The re-evaluations presented above are good examples for demonstrating possible applications of ANCAC. Further studies *i. e.* concerning the abundance of oxygen containing amino acids derived from aerobic versus anaerobic synthetic pathways or the abundance of methionine and cysteine in sulphur anabolic pathways and many more are feasible. Even bias of amino acid composition of proteins differentially expressed during different metabolic states could be detected by ANCAC in batch processing mode. Such bias has already been reported for *Saccharomyces cerevisiae* during oxidative and reductive energy-yielding reactions [15]. Although the COG database has become a standard for ‘uniform-function’ protein groups [16], it contains only 66 representative genomes. The organism coverage of the COG and archaeal COG databases currently implemented into ANCAC is therefore a limitation of the tool.

## Conclusions

Many studies dealing with links between sequence features such as nucleotide, amino acid, and codon frequencies and functional aspects of proteins as well as biological or phylogenetic issues have been published so far. All of them have required intensive programming work since there has been no software-tool to directly and simply perform such computations. ANCAC, although currently limited to the data of the COG and arCOG databases, ultimately fills this gap.

## Availability and requirements

**Project name:** aminoacid, nucleotide, and codon analysis of COGs (ANCAC)

**Project homepage:**http://www.uni-wh.de/ancac

**Operating system(s):** Platform independent

**Programming languages:** Perl, JavaScript, CSS, HTML, and SQL

**Other requirement:** latest web-browser compatible with HTML5 and capable of executing JavaScript

**License:** GNU general public licence

**Any restrictions to use by non-academics:** contact authors

## Abbreviations

ANCAC: Aminao acid, nucleotide, and codon analysis of COGs; APSF: Average percentage of the sequence features selected; arCOG: Archaeal clusters of orthologous genes; arNUCOCOG: Archaeal nucleotide containing clusters of orthologous genes database; CGI: Common gateway interface; COG: Cluster of orthologous groups; CSS: Cascading style sheets; DBI: Database interface; GUI: Graphical user interface; HTTP: Hypertext transfer protocol; NUCOCOG: Nucleotide containing COG database; OGT: Optimum growth temperature; Perl: Practical extraction and report language; SQL: Structured query language; XML: Extensible markup language.

## Competing interests

The authors declare that they have no competing interests.

## Authors’ contributions

AM developed the web-interface and the underlying database, CK conceived of the study and assigned the COGs of all enzymes involved in amino acid anabolism, MK conceived of the study, participated in its design and coordination and drafted the manuscript. All authors read and approved the final manuscript.
